# Resting-State EEG Correlates of Childhood Maltreatment and Depression: Potential Neurophysiological Links and Future Research Directions

**DOI:** 10.3390/neurosci7010003

**Published:** 2025-12-31

**Authors:** Christopher B. Watson, Christopher F. Sharpley, Vicki Bitsika

**Affiliations:** Brain-Behaviour Research Group, University of New England, Armidale, NSW 2351, Australiavicki.bitsika@une.edu.au (V.B.)

**Keywords:** childhood maltreatment, depression, electroencephalography, biomarker

## Abstract

The experience of childhood maltreatment (CM) increases the risk for depressive disorders by two-and-a-half times across the lifespan. Although stress system and immunological models offer some explanation of this vulnerability, further investigation is required to understand the underlying neurophysiological mechanisms and identify potential biomarkers for diagnosis and treatment. Resting-state electroencephalography (EEG) offers a low-cost, non-invasive, and accessible methodology for that purpose. This narrative review synthesizes resting-state EEG findings that are common to CM and depression as a primer for further research and the future formulation of a model that may link these two in a causal manner. Although evidence supports atypical beta and theta band power, frontal alpha asymmetry and altered default mode network functional connectivity as possible indicators of the CM-EEG association, there is a paucity of EEG-based CM research available to complement the extensive depression-focused literature. Large-sample, prospective EEG studies of CM that consider confounding factors and assess the neurophysiological impact of CM independent of psychopathologies are required.

## 1. Introduction

Childhood maltreatment (CM) is the experience of adverse events such as abuse (physical, emotional, and sexual), neglect (physical and emotional), or household instability (e.g., parental substance abuse, exposure to intimate partner violence, parental separation, household mental illness, and parental incarceration) before the age of 18 years [1,2]. Global rates of CM are estimated at up to 25% [3] and are associated with poor long-term health and wellbeing [2]. In their expert review, Teicher et al. [4] described CM as the most important preventable risk factor for the diagnosis, treatment, and prevention of psychiatric disorders. In particular, there is substantial evidence that CM is a risk factor for the development of depressive disorders [5,6], with meta-analytic reviews reporting that individuals with a history of any CM are two-and-a-half times more likely to experience depression in adulthood [7,8]. Depression itself is a significant contributor to the global disease burden and one of the leading causes of disability worldwide [9] with an estimated 12-month global prevalence of 5% [10] and a lifetime incidence rate of up to 30% [11]. It also imposes a substantial economic burden [12] with an estimated loss of 12 billion productive workdays each year and an approximate annual cost approaching USD1 trillion [13].

### 1.1. Possible Pathways Between CM and Depression

The consistent evidence linking CM to depression suggests that there may be stable neurophysiological mechanisms underlying that association. Research has sought to identify these factors for further examination as potential biomarkers for depression [4,14]; however, current findings are inconsistent and have a narrow application to depression biomarker development. For example, dysregulation of the hypothalamic–pituitary–adrenal (HPA) axis has been reported as an important factor in the development of depression [15] and has been shown to arise from CM exposure via hypercortisolemia (after acute stressor events) and possible hypocortisolemia (after ongoing major stressful events that exhaust the HPA axis response) [16]. Yet only a subset of depressed individuals exhibit HPA axis dysregulation [17], and cortisol studies of participants who experienced CM are inconsistent, with some reporting elevated levels [18,19] and others reporting decreased levels [20,21].

Another common hypothesis is that CM causes inflammatory changes that are consistent with the pathophysiology of depressed individuals [22,23]. Researchers propose that the stress of CM generates a prolonged immunoinflammatory response that results in a long-term elevation of cytokines and protein markers and leads to negative physical health outcomes [24]. The neuroimmune network hypothesis of early-life adversity posits that this chronic inflammation interacts with neural circuitries subserving threat, reward, and executive control to increase vulnerability for negative health outcomes such as psychopathology [25]. However, a number of contradictory studies report no relationship between CM and inflammation or report a limited association between specific forms of CM and inflammation [26,27]. Furthermore, while meta-analytic studies report a strong link between inflammation and depression [28,29], it is estimated that only one-quarter of depressed individuals display elevated inflammatory markers, which has led researchers to propose inflammation-linked depression as a subtype of depression [30].

### 1.2. Exploring Underlying Mechanisms Using Electroencephalography

Although both HPA axis and neuroimmune/inflammatory factors may contribute to the association between CM and depression, gaps remain in the understanding of the underlying neurophysiological mechanisms of that relationship. (EEG). EEG is a neuroimaging technique that measures the electrical signals created by the dendritic membrane of pyramidal neurons near the surface of the brain [31,32]. It involves attaching electrodes to the scalp, which record the currents flowing in the extracellular space via a process of volume conduction [31,33]. These signals, which are produced by clusters of excitatory and inhibitory postsynaptic potentials, pass through the brain tissue, skull, flesh, and skin layers to the scalp, where they are amplified to allow recording by a brain–computer interface [31,33].

EEG is an ideal modality for biomarker research because it is low cost, widely available, non-invasive and has a high temporal resolution [34], as well as allowing accurate assessment of intrinsic brain activity that may be associated with specific factors such as CM. In particular, resting-state EEG is a valuable tool to understand the CM–depression connection because it does not require participants to undergo a cognitive task, thereby minimizing performance confounds. Although some previous criticisms of EEG research as a potential biomarker for depression have argued that interpretations are limited to the cortex surface [35], recent advances in signal processing can now provide functional EEG brain-imaging with distinctly improved spatial resolution that complements its already reliable temporal resolution [36].

The purpose of this review is to examine resting-state EEG findings that are similar across studies of depression and CM with the aim of identifying shared abnormal neurophysiological aspects. This kind of review has not been published previously and may act as a primer for the future development of a model for synthesis of two disparate (but possibly connected) studies. It is theorized that mutual correlates (i.e., CM-EEG; depression-EEG) may provide targets for future depression biomarker research and improve the understanding of the association between CM and depressive symptoms. The elucidation of mutual EEG biomarkers for CM and depression has the potential to create a more objective approach to depression diagnosis that improves upon the existing subjective self-report and clinical assessment approaches. Similarly, CM is largely based upon adult self-report of childhood experiences, and an objective (e.g., EEG data) indicator of CM would enhance diagnostic procedures.

A narrative review was selected to synthesize the literature due to the broad nature of the topic, which spans several EEG analytic methodologies. Alternative approaches such as systematic or scoping reviews require narrowly defined research questions and strict inclusion/exclusion criteria, which do not allow the synthesis of diverse study designs. Furthermore, a narrative approach was deemed appropriate after extensive independent literature searches by two researchers (C.B.W. and C.F.S.) identified a disparity between the large number of EEG studies available on depression and the limited number CM-EEG investigations. No previous work has compared the EEG findings of CM and depression studies, and, as such, this narrative review provides a novel approach to identifying targets for biomarker research and provides a primer for future original research projects and subsequent systematic reviews and meta-analyses.

## 2. EEG Frequency Bands

The cortical signal measured by EEG is classified by distinct frequency bands, including delta (1–4 Hz), theta (4–8 Hz), alpha (8–13 Hz), beta (13–30 Hz), and gamma (30–200 Hz), which have a variable power spectral density (i.e., the distribution of power amplitude or signal strength) [37]. Each band is associated with a unique neurophysiological state, and Table 1 shows the classification of brain waves into frequency bands and neurocognitive states, as described by Greco et al. [38].

## 3. Atypical Absolute Power

### 3.1. Depression

Both CM and depression have been linked to altered absolute power (total power within a frequency band) across beta and theta bands during resting-state conditions, and research has proposed this variation as a promising neurobiological marker. In a meta-analysis of 18 depression studies published between 1996 and 2018, Newson and Thiagarajan [39] concluded that there were consistent findings of increased absolute power in the theta and beta bands of depressed individuals when compared to healthy controls. Those outcomes remained statistically significant regardless of whether the participants were resting with eyes open or eyes closed, and the magnitude of the increased power between the two groups was consistent (50%) across the studies. That review noted that the included studies were limited by small sample sizes (which does not allow for the naturally large heterogeneity of human EEG dynamics) and variability within the frequency bands across studies. However, since that review there have been replicated findings of increased theta and/or beta power in depressed individuals [40,41,42,43]. Elevated activity in these bands is a plausible indicator of depression because they are associated with neurophysiological states that are commonly altered in depressive disorders such as emotional processing, concentration, expectancy, and anxiety [44].

### 3.2. CM

Increased beta and theta power has also been found in individuals with a history of CM. In a study of 48 individuals diagnosed with depression and 49 healthy controls, Xia et al. [45] sought to articulate the impact of CM on the emotional and cognitive performance of the depressed cohort by comparing EEG measurements with participant scores on psychological scales and heart rate variability. Examination of resting-state EEG data revealed that the depressed cohort had increased absolute power in the beta, theta, and delta frequency bands. Furthermore, CM assessment showed that incidences of maltreatment were significantly higher in those individuals with increased absolute powers in these frequency bands. Correlational analysis of EEG absolute power values and CM scores revealed that CM was significantly positively associated with beta and delta powers in the mid-brain regions.

A study of 157 Korean adult volunteers by Lee et al. [46] found a relationship between CM, EEG power, inattention, and attention deficit hyperactivity disorder (ADHD). Participants completed the Childhood Trauma Questionnaire (CTQ) to determine their experience of exposure to CM and were subsequently divided into two groups according to whether they scored high (40–83) or low (28–39) on that scale. These results were compared to resting-state EEG measurements (eyes open and eyes closed, for 3 min each) in addition to participant scores on several psychological scales. Analysis of absolute power values showed that the high-CM group had increased beta, delta, and gamma band powers compared to the low-CM group. However, beta was shown to have the most statistically significant increase across all areas of the brain (anterior, middle, and posterior), with the main effect of delta and gamma being limited to one or two regions.

Alper et al. [47] compared resting-state theta power in a sample of 14 individuals with a history of childhood sexual and/or physical abuse and 13 non-abused controls drawn from a residential treatment facility for crack cocaine dependence. Using standardized low-resolution electromagnetic brain tomography (sLORETA) [48] to estimate source generators, those researchers found that the CM cohort had significantly greater theta power in the parahippocampal, fusiform, lingual, posterior cingulate, and insular gyri. Abnormalities in these areas are linked to maladaptive threat processing [49] and emotional dysregulation [50] which may fit with the development of psychopathologies such as depression. However, these findings are yet to be replicated in large-sample studies, and the inclusion of only cocaine-dependent subjects limits the generalizability of the findings.

### 3.3. Comparison

Preliminary findings suggest that increased beta and theta absolute power may be a shared neurophysiological aspect of CM and depression. However, the number of EEG–depression studies far exceeds that of CM-EEG investigations. Further large-sample CM-EEG studies with a longitudinal prospective design are needed to determine if beta and theta power changes are an underlying neurophysiological mechanism of the association between CM and depression.

## 4. Frontal Alpha Asymmetry

### 4.1. Depression

**Figure 1:**
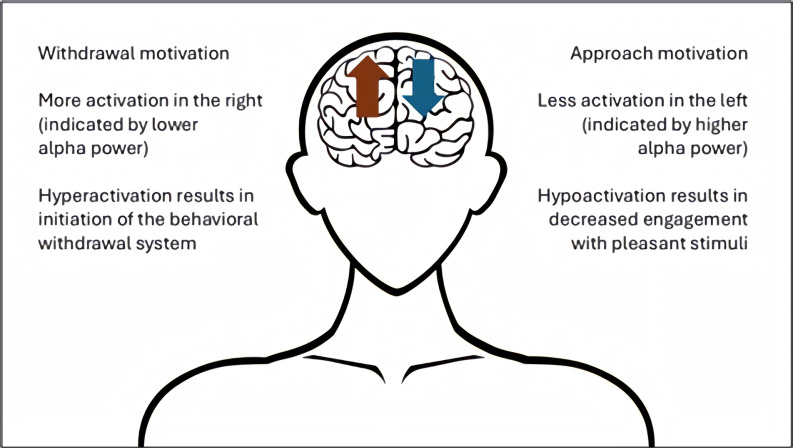
Frontal alpha asymmetry in depression. This figure demonstrates the behavioral withdrawal model of frontal alpha asymmetry in depression where hyperactivation in the right hemisphere results in avoidance of aversive stimuli and hypoactivation in the left hemispheres leads to decreased engagement with pleasant stimuli.

Resting-state frontal alpha asymmetry (FAA) is an EEG measure that indicates differences in the relative alpha wave activity between the left and right frontal cortices of the brain at rest [51]. Several studies have identified stronger right-side frontal activity in depressed individuals when compared to healthy controls and proposed this as a potential diagnostic biomarker [35,52,53,54]. As shown in Figure 1, researchers have hypothesized that this right-side hyperactivation is related to depression as a pathological initiation of the behavioral withdrawal system responsible for avoiding aversive stimuli, while left-side hypoactivation is related to decreased engagement with pleasant stimuli [55,56].

A recent review of 29 depression studies by Xie et al. [51] determined that FAA was a consistent and stable finding. Furthermore, that study found that participants with maternal depression showed greater right asymmetry at rest, which indicated that genetic influences may have an impact on frontal alpha. Researchers have proposed that FAA may play a prognostic role in the severity and chronicity of symptoms [35,57] and serve as a method for distinguishing between depressive disorders [58], with some machine learning models demonstrating up to 85% accuracy in that process [59]. FAA may also contribute significantly to treatment models [60,61]; for example, left-side transcranial alternating current stimulation (tACS) has been proposed as a non-invasive treatment approach for depression based on FAA findings, and early studies have shown promising results [62,63].

### 4.2. CM

FAA has also been proposed as a neurophysiological consequence of CM based on research demonstrating strong links between stress exposure and fluctuations in frontal lobe power and lateralized brain structures [64,65]. As a major life stressor, studies have hypothesized that CM results in long-term and stable changes to frontal alpha activity. In an analysis of 38 maltreated adolescent females and 25 healthy controls, Miskovic et al. [66] examined group differences in resting-state FAA and vagal tone at two timepoints over a six-month period as biological indices of stress vulnerability. Data analysis revealed that at timepoint one, the CM group showed more cortical activity (less alpha power) in the right frontal brain regions and the control group showed more cortical activity in the left frontal region. The CM group also exhibited lower vagal tone than the control group. Analysis at the second timepoint demonstrated that both indices remained stable across the six-month period. As such, those authors argued that, as an EEG correlate of CM, FAA may not simply reflect short-term stress response consequences but long-lasting neurophysiological alterations.

A complicating factor in the existing research of the relationship between CM and FAA is that most investigations have occurred in the context of a specific psychological disorder or symptoms. A limitation of these analyses is that they do not sufficiently establish CM’s contribution to FAA independent of psychopathology. For example, Popkirov et al. [67] sought to determine if CM combined with dissociative symptoms predicted FAA in participants with borderline personality disorder (BPD) before and after the application of a mood induction paradigm. In that study, 26 participants with BPD were paired with 26 healthy controls, and all subjects completed the Childhood Trauma Questionnaire (CTQ) to assess CM exposure and the Dissociative Experiences Scale (DES) to determine dissociative symptoms. The sample undertook eight minutes of resting-state EEG measurement and an additional eight minutes while viewing a mixture of aversive and neutral pictures. Baseline analysis of the resting-state data revealed that FAA correlated significantly with CM in participants with BPD; however, there was insufficient incidence of CM within the control group to determine if it resulted in FAA without the presence of BPD. Both groups showed a shift to right-sided asymmetry after viewing negative images as part of the mood induction test. This suggests a consistent EEG response to adverse experiences between individuals regardless of BPD and provides some support for the CM/FAA hypothesis; however, these results are yet to be replicated and studies that adequately account for confounding psychopathologies are needed.

In a sample of 314 adults, Hostinar et al. [68] examined whether a relationship existed between CM and FAA as a model to explain the interaction between stress, inflammation, and depression. That study gathered resting-state EEG measurements and inflammatory markers and assessed exposure to CM using the CTQ and depression using the 20-item Centre for Epidemiologic Studies Depression (CES-D) inventory. Analysis revealed that CTQ scores were not independently associated with FAA; however, asymmetry was associated with inflammation in participants who reported moderate-to-severe CM. Those authors suggested that some individuals may have a tendency for asymmetry to manifest after severe adversity, which, in turn, predisposes them to inflammation, but further investigation is required.

### 4.3. Comparison

Overall, there is promising evidence that power asymmetry in the alpha frequency is a shared EEG correlate of CM and depression; however, further research is needed to determine the contribution of CM independent of confounding variables. Furthermore, like the literature examining altered frequency band power, there are substantially more published studies of the relationship between depression and FAA than CM-FAA. It is recommended that future research seeks to replicate existing findings from studies of CM and FAA in addition to creating new original research projects.

## 5. Altered DMN Functional Connectivity

The brain is composed of interconnected regions that display patterns of synchronized activity believed to represent unique cognitive, emotional, and behavioral states [69]. EEG allows analysis of the functional connectivity (FC) between these regions through statistical examination of signals from across the various frequencies that are received by electrodes placed at different locations on the scalp [70]. FC analysis provides information about the interaction of brain regions that are not necessarily structurally connected but form complex brain networks [71]. Alterations in resting-state FC have been proposed as reliable markers for the classification and prediction of brain disorders like depression [72] and for identifying the neurophysiological consequences of major life stressors like CM [73].

### 5.1. Depression

**Figure 2:**
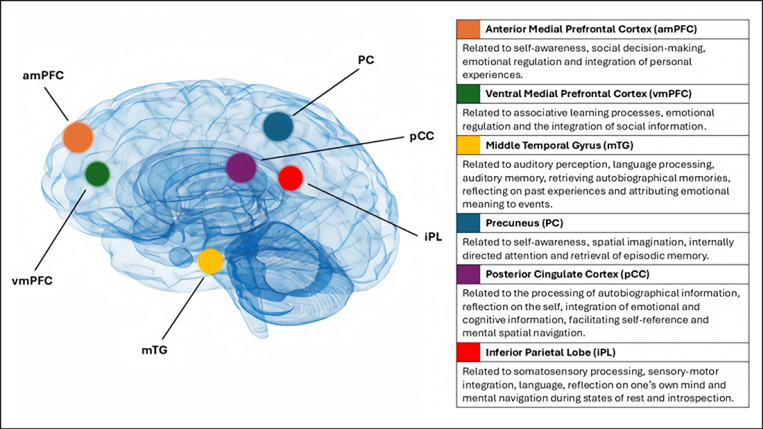
The default mode network—neuroanatomy and functional links. This figure displays the neuroanatomy of the default mode network and the functional associations of each of these areas. Adapted from [76].

A consistent finding in depression research is variation in the FC of the default mode network (DMN) [74]. The DMN is a set of interconnected brain regions that decrease in activity during goal-directed tasks and activate during rest [75]. As shown in Figure 2, the network consists of the anterior and ventral medial prefrontal cortex, the posterior cingulate cortex, the precuneus, the inferior parietal lobe, and the middle temporal gyrus [76]. These areas are implicated in functions that are commonly disturbed in depressed individuals, such as self-referential mental activity, affective processing, rumination, and the formation of internal emotional states [77,78,79].

Resting-state EEG studies of depressed cohorts have identified alterations to the overall FC within the DMN. A study by Ho et al. [80] examined group differences in DMN connectivity between individuals with major depressive disorder (MDD) and healthy control groups using EEG coherence as an index of FC. That study identified increased high-beta-frequency (25–30 Hz) FC within the DMN and proposed that this hyper-functional connectivity indicates that MDD sufferers have trouble processing self-referential or negative autobiographic memories. Similarly, Choi et al. [81] found depressed individuals showed global DMN hyperactivation in the upper beta range (18–30 Hz) compared to healthy controls.

EEG investigations have also identified altered connectivity between the DMN and other major neural networks in depressed cohorts. A study by Knyazev et al. [82] reviewed the FC between DMN and task-positive and task-negative networks in a sample of 41 individuals with MDD and 23 controls. That study found increased FC between the DMN and major emotion and attention regulation circuits in the MDD cohort compared to healthy controls. The authors argued that their findings suggested an increased readiness to respond to self-related thoughts over environmental stimuli. In a study of 65 individuals with MDD and 79 healthy controls, Whitton et al. [83] identified increased resting-state FC between the DMN and the frontoparietal network (FPN) in the MDD cohort. Those authors theorized that, because the FPN is implicated in the inhibition of the DMN during task performance, this increased FC may result in issues synonymous with depression, such as difficulties shifting attention away from self-referential thoughts and rumination to the external stimuli.

### 5.2. CM

Like depression, exposure to CM has been linked to alterations in the overall FC of the DMN and to abnormal connectivity between the DMN and other brain networks. However, the majority of studies have employed functional magnetic resonance imaging (fMRI) to assess FC rather than EEG. fMRI findings include decreased overall DMN connectivity [84], reduced connectivity between the DMN and the salience network [85], and increased FC between the medial prefrontal cortex and the amygdala [86], a neural pathway that plays an important role in the processing of threats and negative emotions [87].

While few resting-state EEG studies have been published reviewing CM and the FC of the DMN, preliminary results from examinations of sub-regions of the DMN appear consistent with the above fMRI findings. A study of 1652 adolescents by Neale et al. [88] assessed the relationship between childhood trauma, neurodevelopment, alcohol use disorder, and post-traumatic stress disorder in frontal sites within the alpha frequency. That study determined that childhood sexual-assaultive trauma was associated with higher EEG coherence in the left frontocentral and interhemispheric prefrontal regions of female participants, while physical-assaultive trauma was linked to lower coherence in those regions in males. An earlier study by Black et al. [89] comparing EEG connectivity patterns in 24 adults with a history of childhood sexual abuse and 24 controls without abuse exposure found decreased alpha and beta connectivity in right frontal and posterior midline regions and increased connectivity centrally and in the left temporal lobe in the delta frequency.

### 5.3. Comparison

These findings, plus the outcomes of fMRI research, suggest that it is likely that CM is associated with altered development of the DMN, and that this alteration may be a neurophysiological link to depression. CM-related stress exposure may alter the developing brain in a way that is expressed as DMN hypo-connectivity in the short term and hyper-connectivity in the long term, resulting in changes to emotion regulation and self-referential thought processing in individuals with heightened vulnerability for depression. However, further EEG research examining the FC of the DMN as a whole and its connectivity with other major neural networks is required.

## 6. Conclusions

There is consistent empirical evidence that CM is an important preventable risk factor for the development of depressive disorders. Despite this, there is limited understanding of the underlying mechanisms of that association. Existing investigations of CM’s neurophysiological impact have focused on stress response dysregulation and immunoinflammatory alterations as mechanisms for the development of psychopathology. However, only a subset of depressed individuals exhibits HPA axis dysregulation and inflammatory responses. As an alternative pathway to understanding the CM–depression relationship, resting-state EEG provides promising evidence that CM and depression may share neurophysiological correlates that have the potential for future biomarker research.

This review examined atypical beta and theta frequency band power, FAA, and altered DMN FC as promising EEG links for further examination. Increased beta and theta absolute power values were reported in EEG studies of both depression and CM; however, there were substantially more depression studies available than CM-EEG investigations, making direct comparisons difficult. Further large-sample CM-EEG studies are required that consider the neurophysiological impact of CM independent of confounding variables and psychopathologies. FAA was well supported as an indicator of depression, with meta-analytic studies arguing that it has viable diagnostic utility to complement existing clinical assessment approaches. By comparison, the evidence of CM’s association with FAA is growing, but further original research and replication of existing findings is needed. A future pathway for research may be to test the approach and withdrawal hypothesis of depression–FAA whereby the chronic stress of CM initiates FAA and the behavioral withdrawal symptoms synonymous with depression. Finally, there were consistent reports of DMN FC alteration in both depression and CM research, although depression studies have reported increased FC while CM investigations have determined decreased connectivity. Nevertheless, the reviewed evidence suggests that alterations to FC, both within the DMN and between the DMN and other neural networks, are a shared EEG finding of CM and depression. Future research should employ consistent FC measurement approaches with particular focus on upper beta frequency ranges.

This is the first narrative review to compare resting-state EEG findings from CM and depression studies for the purposes of identifying common biomarker research targets. Despite some initial findings from EEG-CM research, the field is currently under-represented compared to EEG investigations of depression, therefore hindering the elucidation of common EEG-related pathways between CM and depression. This imbalance of empirical evidence made a systematic approach to the current review inappropriate. Future research can improve the understanding of the association between CM and depression by expanding the EEG analysis of CM to include large-sample longitudinal prospective studies. This will allow a more balanced review of the literature with well-defined research questions and a replicable systematic methodology.

The scope of this review was limited to examining depression and CM as homogenous concepts for the purpose of providing a preliminary assessment of neurophysiological links. Future research should also consider the subtypes of depression and CM to determine if plausible links are moderated by specific symptoms of depression (e.g., anhedonia, somatic) or forms of CM (e.g., sexual abuse, emotional neglect). Furthermore, confounding factors and comorbidities of depression, such as treatment effects and post-traumatic stress disorder, should be examined. Based on the current review, it is proposed that atypical beta and theta frequency band power, FAA, and altered DMN FC warrant further investigation as potential neurophysiological links between CM and depression that may improve understanding of the underlying mechanisms of that association.

## Figures and Tables

**Table 1:** Classification of EEG brain waves.

Brain Waves	Frequency Range (Hz) *	Neurophysiological Association
Delta	1–4	deep sleep
Theta	4–8	meditation, deep relaxation
Alpha	8–13	resting, dormancy, relaxed wakefulness, inhibitory activity
Beta	13–30	conscious thought, withdrawn concentration, anticipation
Gamma	30–200	attention, higher-order thinking

* These ranges are subject to some variability across individuals.

## Data Availability

No new data were created or analyzed in this study. Data sharing is not applicable to this article.

## Abbreviations

The following abbreviations are used in this manuscript:

amPFC	(Anterior Medial) Prefrontal Cortex
BPD	Borderline Personality Disorder
CES-D	Centre for Epidemiologic Studies Depression
CM	Childhood Maltreatment
CTQ	Childhood Trauma Questionnaire
DES	Dissociative Experiences Scale
DMN	Default Mode Network
EEG	Electroencephalography
FAA	Frontal Alpha Asymmetry
FC	Functional Connectivity
fMRI	(Functional) Magnetic Resonance Imaging
FPN	Frontoparietal Network
HPA	Hypothalamic–Pituitary–Adrenal
iPL	(Inferior) Parietal Lobe
MDD	Major Depressive Disorder
mTG	(Middle) Temporal Gyrus
PC	Precuneus
pCC	Posterior Cingulate Cortex
sLORETA	Standardized Low-Resolution Electromagnetic Brain Tomography
tACS	(Transcranial) Alternating Current Stimulation
vmPFC	(Ventral Medial) Prefrontal Cortex
